# Cerebrospinal Fluid Lactate Levels as a Prognostic Indicator in Patients With Cryptococcal Meningitis Who Are HIV Negative: A Retrospective Cohort Study

**DOI:** 10.1093/ofid/ofae540

**Published:** 2024-09-20

**Authors:** Yu-Chi Tsai, Yao-Shen Chen, Cai-Sin Yao, Ren-In Chang, Ning-Chi Wang, Jui-Kuang Chen

**Affiliations:** Division of Infectious Diseases, Department of Internal Medicine, Kaohsiung Armed Forces General Hospital, Kaohsiung, Taiwan; Division of Infectious Diseases and Tropical Medicine, Department of Internal Medicine, Tri-Service General Hospital, National Defense Medical Center, Taipei, Taiwan; Division of Infectious Diseases, Department of Internal Medicine, Kaohsiung Veterans General Hospital, Kaohsiung, Taiwan; Department of Administration, Kaohsiung Veterans General Hospital, Kaohsiung, Taiwan; Faculty of Medicine, School of Medicine, National Yang Ming Chiao Tung University, Taipei, Taiwan; Department of Business Management, National Sun Yat-Sen University, Kaohsiung, Taiwan; Department of Medical Education and Research, Kaohsiung Veterans General Hospital, Kaohsiung, Taiwan; Department of Medical Education and Research, Kaohsiung Veterans General Hospital, Kaohsiung, Taiwan; Department of Emergency Medicine, Veterans General Hospital, Kaohsiung, Taiwan; Institute of Medicine, Chung Shan Medical University, Taichung, Taiwan; Division of Infectious Diseases and Tropical Medicine, Department of Internal Medicine, Tri-Service General Hospital, National Defense Medical Center, Taipei, Taiwan; Division of Infectious Diseases, Department of Internal Medicine, Kaohsiung Veterans General Hospital, Kaohsiung, Taiwan; School of Nursing, Fooyin University, Kaohsiung, Taiwan; School of Medicine, National Defense Medical Center, Taipei, Taiwan; School of Medicine, College of Medicine, National Sun Yat-sen University, Kaohsiung, Taiwan

**Keywords:** cerebrospinal fluid lactate, cryptococcal meningitis, human immunodeficiency virus, retrospective review, Taiwan

## Abstract

**Background:**

Cryptococcal meningitis (CM) is a severe central nervous system infection. In patients with HIV infections and coexisting CM, elevated baseline cerebrospinal fluid (CSF) lactate levels can predict increased mortality. However, the CSF lactate level's significance in patients with CM who are HIV negative remains unclear, necessitating further investigation to elucidate the potential distinctions and enhance patient management. This study investigated the significance of CSF lactate levels in patients with CM who were HIV negative.

**Methods:**

This retrospective study utilized data from the clinical databases of patients who underwent lumbar punctures at a medical center in Kaohsiung City, southern Taiwan. Demographic data, CSF lactate levels, routine CSF analyses, and hematologic and neurologic findings were evaluated. The optimal CSF lactate threshold value was determined by the Youden index.

**Results:**

This retrospective study included 70 patients with CM, among whom 44 (63%) and 26 (37%) tested negative and positive for HIV, respectively. The group without HIV exhibited higher CSF lactate levels, with an optimal CSF lactate cutoff point of 7.935 mmol/L for predicting 90-day mortality, resulting in significant predictive accuracies (area under the curve, 0.755; sensitivity, 57.1%; specificity, 100%); this value was an independent mortality predictor in patients who were HIV negative. In patients with CM who were HIV negative, CSF lactate levels ≥7.935 mmol/L correlated with higher mortality rates but without statistical significance. All patients with CM who were HIV negative and had CSF lactate levels ≥7.935 mmol/L died within 3 months of admission.

**Conclusions:**

Patients with CM who were HIV negative had elevated CSF lactate levels that correlated with adverse outcomes, enabling early identification of high-risk individuals.

## Teaser

Elevated cerebrospinal fluid lactate levels are a potential early risk marker to predict higher mortality in patients with cryptococcal meningitis who are HIV negative. Further validation studies are necessary.

Cryptococcal meningitis (CM) is the most prevalent fungal infection affecting the central nervous system [1]. Historically, it occurred only in people with HIV and AIDS; however, recent studies have revealed an expansion in CM's spectrum of vulnerability. High-risk groups now include immunocompromised populations, such as organ transplant recipients, patients with cancer undergoing antineoplastic therapy, and those using immunosuppressants [2]. Although the clinical presentation may vary across diverse populations, the unchanging reality is the substantial morbidity and elevated mortality rates associated with CM, imposing a significant global public health burden [3, 4]. Thus, early identification of high-risk patients is crucial in guiding clinicians’ therapeutic choices during this pivotal phase of the disease trajectory.

In managing CM among patients with HIV, a critical determinant linked to increased mortality is a baseline cerebrospinal fluid (CSF) lactate level exceeding 5 mmol/L [5]. This discovery accentuates the pivotal role of CSF lactate level monitoring in individuals concurrently battling HIV and CM, providing an invaluable prognostic gauge that can guide treatment decisions.

Conversely, in patients with CM who are HIV negative, the role of CSF lactate levels remains unclear, prompting investigations into the underlying pathophysiologic differences between patients with CM who are HIV positive and HIV negative. Therefore, we aimed to examine the significance of CSF lactate levels in patients with CM who are HIV negative to enhance our capacity to address this formidable condition and improve patient outcomes.

## METHODS

This retrospective study utilized data from the clinical databases of a medical center in Kaohsiung City, southern Taiwan. The data set comprised demographic information (eg, age and sex), medical histories, reasons for admission, postadmission tests and treatments, prescribed medications, and discharge diagnoses. Diseases were classified per the *International Classification of Diseases*, *Ninth Revision* and *Tenth Revision* (*ICD-9* and *ICD-10*).

### Study Population

Patients were included in the study if they were adults (≥18 years) who were hospitalized for CM between January 1991 and March 2023 and underwent lumbar puncture with available CSF lactate data. Patients with CM were identified by *ICD* codes: CM (*ICD-9*, 312.0), cerebral cryptococcosis (*ICD-10*, B45.1), and unspecified cryptococcosis (*ICD-10*, B45.9).

The exclusion criteria were individuals lacking CSF lactate data or cases in which complete records were discarded because they exceeded the legal retention period, resulting in insufficient clinical analysis and identification data. CM was defined as the isolation of *Cryptococcus* species from a CSF specimen, a positive CSF cryptococcal antigen test result, or a positive CSF India ink test result [6].

### Laboratory Data Measurement and Imaging Assessment

All study participants underwent lumbar punctures as part of the diagnostic evaluation for CM. CSF lactate levels were quantified with a lactate analyzer (Automated Chemistry Analyzer; Beckman Coulter). In addition to CSF lactate measurement, routine CSF analyses included a comprehensive assessment of white blood cells (WBCs) and differential counts, glucose levels, protein concentrations, India ink staining, and microbial cultures. Hematologic evaluations, including screening and confirmatory testing for HIV infection, were conducted with a sensitive enzyme immunoassay kit (Cobas 4800 Systems; Roche).

Brain imaging, such as computed tomography or magnetic resonance imaging, was performed when clinically indicated, and the findings were meticulously reviewed. Neuroimaging findings such as meningeal enhancement, cerebral infarctions, hydrocephalus, abscesses, and other relevant manifestations were evaluated.

### Protocols for the Treatment of CM

All patients received antifungal agents for a minimum of 1 week, with treatment options including amphotericin B, fluconazole, or flucytosine. Supplementary Table 1 provides a detailed breakdown of patient treatment regimens.

In cases where patients presented with clinical signs indicative of increased intracranial pressure or CSF opening pressures exceeding 250 mm H_2_O during their initial lumbar puncture upon admission, therapeutic interventions were implemented to mitigate elevated intracranial pressures [7, 8]. These interventions included sequential repeated lumbar punctures and temporary or permanent intraventricular drainage.

The survival status and neurologic conditions of the patients were meticulously recorded at days 30, 60, and 90 and 1 year after hospital admission. An adverse neurologic status was defined as a modified Rankin scale score ≥2.

### Study Outcomes and Analytical Approach

Given that the mortality rate of patients with CM in the first 3 months after infection is close to 20% [9], with the majority of deaths occurring in the first month [10], the primary end point of this study was the rate of all-cause mortality at specific time points: 30, 60, and 90 days after hospital admission. The secondary outcome measures were the proportion of patients who underwent temporary or permanent intraventricular shunting, duration of antifungal agent use, length of hospital stay, the CSF culture's sterile time, and those who had unfavorable neurologic outcomes. Variables associated with adverse prognoses were included in the univariate and multivariate analyses.

### Statistical Analyses

Demographic and clinical characteristics were summarized for the groups of individuals with CM, with and without HIV, and presented as mean ± SD or number (percentage), depending on the nature of the data. Continuous variables were compared by *t* test when examining baseline characteristics, whereas categorical variables were assessed by chi-square or Fisher exact test. Cox regression analysis was used to identify the independent risk factors associated with 90-day mortality. Variables with *P* values <.05 in the univariate analysis were included in the multivariate Cox regression model. CSF lactate levels were used to evaluate the predictive accuracy for 90-day mortality, and receiver operating characteristic analysis was conducted, including calculation of sensitivity and specificity as well as area under the curve. The optimal threshold value for CSF lactate was determined with the Youden index, which was used to categorize CSF lactate levels into high- and low-value groups. Unadjusted survival curves were generated via the Kaplan-Meier method, and the 2 sets of curves were compared by the log-rank test. All statistical analyses were performed with Stata version 17.0 (StataCorp). Statistical significance was defined as *P* < .05.

### Ethical Statements

Ethical approval for this study was obtained from the Ethics Committee of the Kaohsiung Veterans General Hospital (KSVGH22-CT1-02). This study was conducted in adherence with the principles outlined in the Declaration of Helsinki and the guidelines of the International Conference on Uniform Clinical Medicine Standards to ensure the complete protection of patient privacy. In light of the retrospective design and the absence of prospectively collected data and interventions or interactions with participants, the requirement for obtaining written informed consent was waived.

## RESULTS

**Figure 1.**
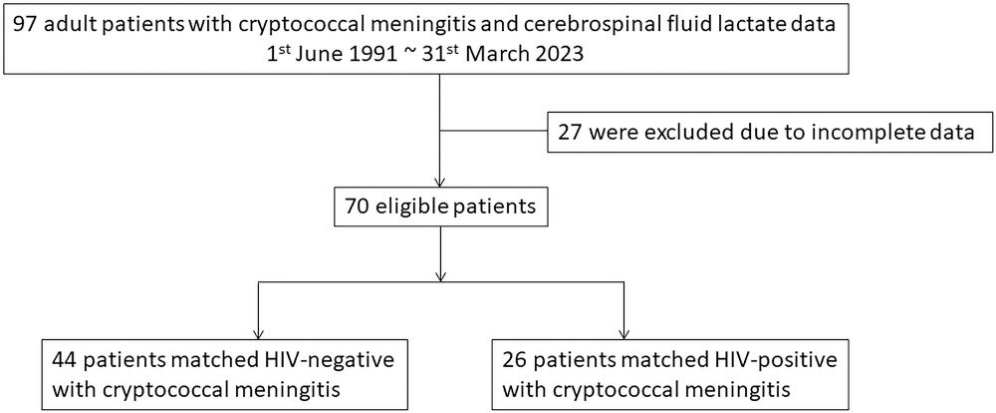
Flowchart of the patient selection process.

A total of 97 adult patients with *ICD-9* code 312.0 and *ICD-10* codes B45.1 and B45.9 between 1991 and 2023 were enrolled in this study. Among them, 27 with missing information in their medical records were excluded. The remaining 70 patients with CM were included in the analysis: 44 (63%) who were HIV negative and 26 (37%) who were HIV positive (Figure 1).

Table 1 provides an overview of the patients included in this study (mean age, 54.1 years; male, 60%). Patients with CM who were HIV negative were older (62.3 ± 18.8 vs 40.2 ± 10.5 years, *P* < .001) and had a lower proportion of male individuals (45.5% vs 84.6%, *P* = .002) than patients with CM who were HIV positive. Additionally, they had higher incidences of hypertension (40.9% vs 7.7%, *P* = .003) and rheumatic disease (18.2% vs 0%, *P* = .022). In general, the average Charlson Comorbidity Index was 4.7 ± 2.6. The median time from symptom onset to CM diagnosis was 11.5 days (IQR, 7–30). Except for a lower frequency of blurred vision complaints among patients who were HIV negative (0% vs 23.1%, *P* = .002), no significant difference in initial symptom presentation was observed between the groups.

**Table 1.** Patient Demography and Clinical Features

Variable	Total (n = 70)	HIV Negative With CM (n = 44)	HIV Positive With CM (n = 26)	*P* Value
Age, y	54.1 ± 19.4	62.3 ± 18.8	40.2 ± 10.5	**<.001**
Sex: male	42 (60)	20 (45.5)	22 (84.6)	.**002**
BMI	21.9 ± 3.8	22 ± 4.2	21.7 ± 3.3	.749
Hypertension	20 (28.6)	18 (40.9)	2 (7.7)	.**003**
Coronary artery disease	2 (2.9)	1 (2.3)	1 (3.9)	>.999
Cerebral vascular accident	4 (5.7)	3 (6.8)	1 (3.9)	>.999
Dementia	2 (2.9)	2 (4.6)	0	.526
Diabetes mellitus	14 (20)	11 (25)	3 (11.5)	.225
Chronic kidney disease	3 (4.3)	3 (6.8)	0	.289
End-stage renal disease	3 (4.3)	3 (6.8)	0	.289
Cirrhosis	3 (4.3)	3 (6.8)	0	.289
Malignancy	6 (8.6)	6 (13.6)	0	.078
Rheumatic disease	8 (11.4)	8 (18.2)	0	.**022**
History of transplantation	2 (2.9)	2 (4.6)	0	.526
CCI	4.7 ± 2.6	3.5 ± 2.6	6.7 ± 1	**<**.**001**
CD4 count	…	…	47.4 ± 65.8	
HIV viral load	…	…	119 767.1 ± 179 271.9	
Days from initial symptoms to diagnosis, median (IQR)	11.5 (7–30)	10 (5.5–30)	13.5 (7–30)	.805
Fever	37 (52.9)	20 (45.5)	17 (65.4)	.107
Headache	39 (55.7)	23 (52.3)	16 (61.5)	.451
Neck stiffness	19 (14.3)	6 (13.6)	4 (15.4)	>.999
Nausea/vomiting	26 (37.1)	14 (31.8)	12 (46.2)	.230
Dizziness/vertigo	27 (38.6)	16 (36.4)	11 (42.3)	.622
Seizure	7 (10)	4 (9.1)	3 (11.5)	>.999
Blurred vision	6 (8.6)	0	6 (23.1)	.**002**
Diplopia	4 (5.7)	3 (6.8)	1 (3.9)	>.999
Cough	18 (25.7)	9 (20.5)	9 (34.6)	.190
Altered sensorium	28 (40)	20 (45.5)	8 (30.8)	.226
Hearing loss	2 (2.9)	2 (4.6)	0	.526
Unsteady gait	16 (22.9)	10 (22.7)	6 (23.1)	.973
Urine incontinence	5 (7.1)	3 (6.8)	2 (7.7)	>.999
Weight loss	11 (15.7)	7 (15.9)	4 (15.4)	>.999

Data are presented as mean ± SD or No. (%) unless noted otherwise. Bold indicates *P* < .05.

Abbreviations: BMI, body mass index; CCI, Charlson Comorbidity Index; CM, cryptococcal meningitis.

**Figure 2.**
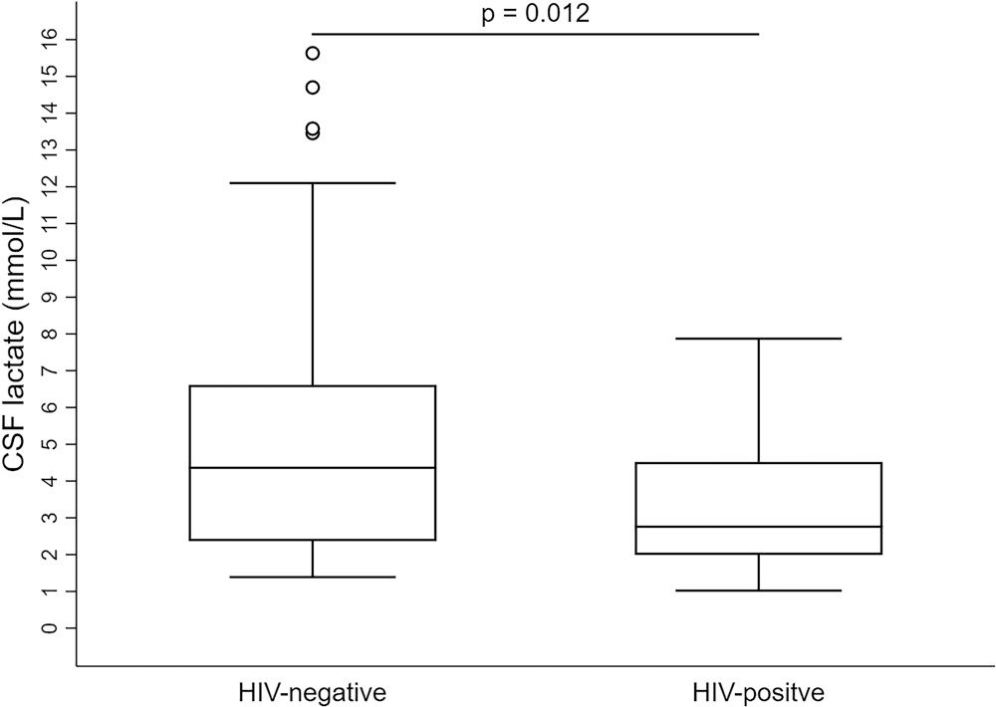
CSF lactate levels in patients with HIV and those without HIV. Line, mean; box, SD; error bars, 95% CI. CSF, cerebrospinal fluid.

When compared with the patients with HIV, those without HIV had higher WBC counts (8.6 ± 3.7 vs 5.8 ± 2.3, *P* < .001) and a lower rate of fungemia (26.6% vs 53.9%, *P* = .044; Table 2). In the CSF analysis, the CSF opening pressure, with an overall mean of 251.2 ± 115.4 mm H_2_O, did not significantly differ between the groups. As compared with patients with HIV, those without HIV had higher CSF WBC counts (125.7 ± 173.5 vs 51.5 ± 84, *P* = .045) and higher proportions of neutrophils and monocytes. Additionally, they had a lower CSF-serum glucose ratio (26.3 ± 17.4 vs 43 ± 19.8, *P* < .001), higher levels of CSF total protein (245.6 ± 332.3 vs 108.8 ± 145.5, *P* = .021), and higher CSF lactate levels (5.4 ± 3.9 vs 3.3 ± 1.9, *P* = .012; Figure 2). The overall rates of CSF India ink positivity and CSF culture positivity were 77.1% and 82.9%, respectively, with no significant differences in radiologic findings between the groups (Supplementary Table 2).

**Table 2.** Laboratory Data Findings

Variable	Total (n = 70)	HIV Negative With CM (n = 44)	HIV Positive With CM (n = 26)	*P* Value
Blood test				
WBC, 10^3^/μL	7.6 ± 3.5	8.6 ± 3.7	5.8 ± 2.3	**<**.**001**
Hemoglobin, g/dL	11.6 ± 2.4	11.3 ± 2.4	12.1 ± 2.5	.225
Platelet, 10^3^/μL	208.1 ± 86.4	199.3 ± 88.7	223 ± 81.9	.270
GOT, U/L (n = 68)	43.3 ± 101	45 ± 123.6	40.4 ± 41.2	.859
GPT, U/L	39.7 ± 71.5	39.5 ± 84.7	40 ± 42.3	.979
Total bilirubin, mg/dL (n = 50)	0.7 ± 0.5	0.8 ± 0.6	0.5 ± 0.3	.**009**
BUN, mg/dL (n = 56)	22.6 ± 23.3	27.4 ± 28	15.1 ± 9.5	.051
Creatinine, mg/dL (n = 69)	1.3 ± 1.7	1.5 ± 2.1	1 ± 0.4	.139
Sodium, mmol/L	136.1 ± 5.4	134.9 ± 5.6	138 ± 4.5	.**014**
Potassium, mmol/L	3.8 ± 0.9	3.9 ± 1.1	3.6 ± 0.5	.228
CRP, mg/dL	2.8 ± 3.8	3.6 ± 4.7	1.8 ± 1.7	.130
Serum lactate, mmol/L (n = 28)	2.0 ± 1.5	1.8 ± 1.2	2.4 ± 2.0	.293
Serum CrAg >1:512 (n = 66)	35 (53)	20 (47.6)	15 (62.5)	.244
Fungemia	27 (38.6)	13 (26.6)	14 (53.9)	.**044**
Lumbar puncture study				
Open pressure, mm H_2_O	251.2 ± 115.4	252.4 ± 109.9	249.1 ± 126.3	.916
Open pressure >250 mm H_2_O	24 (34.3)	15 (34.1)	9 (34.6)	.964
CSF WBC, /μL	98.1 ± 150.4	125.7 ± 173.5	51.5 ± 84	.**045**
CSF Neutrophil, %	13.9 ± 26.8	21 ± 31.6	1.9 ± 4.2	**<**.**001**
CSF Lymphocyte, %	56.3 ± 41.9	58.3 ± 39.1	52.9 ± 46.9	.621
CSF Monocyte, %	6.9 ± 14.1	9.3 ± 16.9	2.8 ± 5.9	.**024**
CSF-serum glucose ratio, %	32.5 ± 20	26.3 ± 17.4	43 ± 19.8	**<**.**001**
CSF Glucose, mg/dL	47.2 ± 34.3	41.3 ± 33.4	57.1 ± 34	.065
CSF Total protein, mg/dL	194.8 ± 284.5	245.6 ± 332.3	108.8 ± 145.5	.021
CSF Lactate, mmol/L^a^	4.6 ± 3.4	5.4 ± 3.9	3.3 ± 1.9	.**012**
CSF Positive India ink	54 (77.1)	35 (79.6)	19 (73.1)	.533
CSF CrAg >1:512	45 (68.2)	30 (71.4)	15 (62.5)	.454
CSF Positive culture	58 (82.9)	37 (84.1)	21 (80.8)	.722

Data are presented as mean ± SD or No. (%). Bold indicates *P* < .05.

Abbreviations: BUN, blood urea nitrogen; CM, cryptococcal meningitis; CrAg, cryptococcal antigen; CRP, C-reactive protein; CSF, cerebrospinal fluid; GOT, glutamic oxaloacetic transaminase; GPT, glutamic pyruvic transaminase; WBC, white blood cell.

^a^The reference range for CSF lactate in this study was <2.8 mmol/L.

Table 3 presents the study outcomes, revealing a lower rate of antifungal consolidation therapy for >8 weeks in the patients with CM who were HIV negative (43.2% vs 80.8%, *P* = .002). Total antifungal use duration was shorter in the patients without HIV (136.9 ± 150.7 vs 332.8 ± 335.2 days, *P* = .007). There were no significant differences between the groups in ventricular shunt placement rates, mortality rates (30, 60, and 90 days), length of hospital stay, CSF culture sterile time, and 1-year adverse neurologic outcomes.

**Table 3.** Study Outcomes by Group

Variable	Total (n = 70)	HIV Negative With CM (n = 44)	HIV Positive With CM (n = 26)	*P* Value
Temporary ventricular shunting	5 (7.1)	4 (9.1)	1 (3.9)	.644
Ventricular peritoneal shunt	11 (15.7)	8 (18.2)	3 (11.5)	.521
Antifungal				
Induction >2 wk	58 (82.9)	36 (81.8)	22 (84.6)	.764
Consolidation >8 wk	40 (57.1)	19 (43.2)	21 (80.8)	**.002**
Total antifungal duration, d	209.6 ± 252.9	136.9 ± 150.7	332.8 ± 335.2	.**007**
Length of hospital stay, d	37.0 ± 24.6	40.6 ± 26.1	30.8 ± 20.7	.081
CSF culture sterile				
>2 wk (n = 58)	13 (22.4)	7 (18.9)	6 (28.6)	.397
Time, d (n = 58)	21.5 ± 34.2	13.1 ± 8.5	27.1 ± 43.2	.280
Death				
Day 30	9 (12.9)	7 (15.9)	2 (7.7)	.468
Day 60	15 (21.4)	12 (27.3)	3 (11.5)	.121
Day 90	19 (27.1)	15 (34.1)	4 (15.4)	.129
Neurologic sequelae^a^	12 (23.5)	9 (31)	3 (13.6)	.147

Data are presented as mean ± SD or No. (%). Bold indicates *P* < .05.

Abbreviations: CM, cryptococcal meningitis; CSF, cerebrospinal fluid.

^a^Neurologic sequelae were defined as patients who survived 12 months after diagnosis with a modified Rankin scale score ≥2.

**Figure 3.**
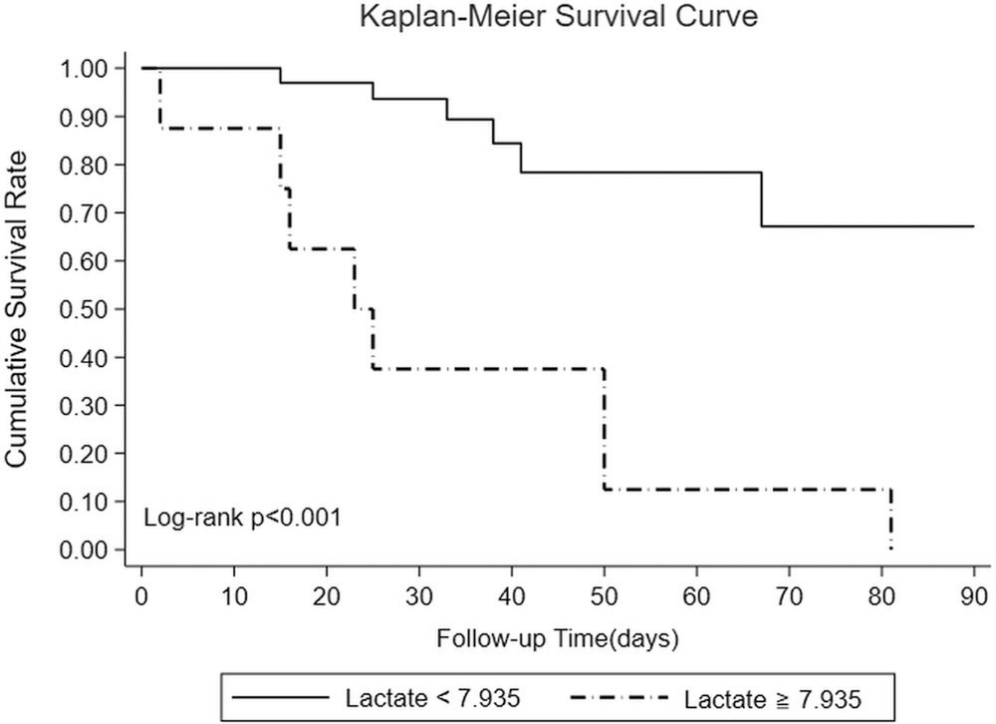
Mortality at 90 days stratified by baseline cerebrospinal fluid lactate level in the group without HIV.

Considering the higher CSF lactate levels observed in the HIV-negative group, attempts were made to identify an optimal CSF lactate cutoff point for predicting 90-day mortality (Table 4). Setting the CSF lactate cutoff at 7.935 mmol/L (area under the curve, 0.755) resulted in a sensitivity of 57.1%, specificity of 100%, positive predictive value of 100%, negative predictive value of 83.3%, and accuracy of 86.4%. CSF lactate levels ≥7.935 mmol/L remained an independent predictor of 30-, 60-, and 90-day mortality rates in patients without HIV in the univariate regression analysis but failed to achieve significance in the multivariate regression analysis of 90-day mortality only (Table 5). In patients without HIV, those with CSF lactate levels ≥7.935 mmol/L had a higher 90-day mortality rate than patients with lower lactate levels (*P* < .001), with all deaths occurring within the first 3 months (Figure 3).

**Table 4.** Receiver Operating Characteristic for CSF Lactate for Prediction of 90-Day Mortality in Patients Without HIV

Day 90 Death	
CSF lactate cutoff, mmol/L	7.935
AUC	0.755
*P* value	.007
95% CI	.58–.93
Sensitivity, %	57.1
Specificity, %	100
PPV, %	100
NPV, %	83.3
Accuracy, %	86.4

Abbreviations: AUC, area under curve; CSF, cerebrospinal fluid; NPV, negative predictive value; PPV, positive predictive value.

**Table 5.** Univariate and Multivariate Cox Proportional Hazard Models for Patients Without HIV

	Day 30 Mortality						Day 60 Mortality						Day 90 Mortality					
	Univariate			Multivariate			Univariate			Multivariate			Univariate			Multivariate		
	HR	95% CI	*P* Value	aHR	95% CI	*P* Value	HR	95% CI	*P* Value	aHR	95% CI	*P* Value	HR	95% CI	*P* Value	aHR	95% CI	*P* Value
CSF lactate ≥7.935 mmol/L	13.98	2.7–72.5	.**002**	1127.90	4.98–255 206	**.011**	7.29	2.29–23.19	.**001**	37.07	1.36–1004.17	.**032**	6.83	2.34–19.90	.**001**	20.15	.84–484.67	.064
Age	1.03	.99–1.07	.142	…			1.02	.99–1.05	.131	…			1.02	.10–1.05	.097	…		
Sex: male	0.18	.02–1.53	.118	…			0.23	.51–1.08	.062	…			0.31	.09–1.13	.075	…		
CCI	1.22	.95–1.56	.114	…			1.14	.94–1.38	.187	…			1.21	1.02–1.44	.**032**	1.009	.80–1.27	.935
Hypertension	1.67	.37–1.47	.503	…			1.02	.33–3.21	.969	…			0.98	.34–2.84	.975	…		
Diabetes mellitus	3.71	.83–16.6	.086	…			2.11	.66–6.71	.207	…			2.57	.86–7.61	.089	…		
Cirrhosis	5.15	.99–26.6	.051	…			2.79	.61–12.79	.187	…			3.92	.52–10.80	.264	…		
Seizure	4.92	.95–25.5	.058	…			3.92	.81–19.07	.090	…			3.92	.81–19.07	.090	…		
Altered sensorium	8.15	.98–67.8	.052	…			6.21	1.36–28.42	.**019**	4.06	.62–26.53	.143	7.24	1.62–32.44	.**010**	5.28	.85–32.73	.074
Malignancy	2.49	.48–12.9	.275	…			3.88	1.15–13.04	.**029**	3.28	.77–13.97	.107	4.83	1.56–14.95	.**006**	3.14	4.59–190.76	.**112**
IICP	0.67	.12–3.31	.597	…			0.96	.30–3.05	.944	…			1.29	.45–3.72	.633	…		
Induction >2 wk	0.14	.04–.54	.**004**	0.08	.006–.93	**.044**	0.25	.08–.82	.**021**	0.29	.05–1.51	.140	0.25	.08–0.82	.**021**	0.26	.05–1.45	.124
Consolidation >8 wk	1.85e-17	…	…	…			0.05	.007–.41	.**005**	0.19	.02–1.79	.146	0.05	.006–.35	.**003**	0.11	.01–1.05	.055
Serum lactate	1.60	1.04–2.46	.**031**	2.25	1.04–4.86	**.039**	1.34	.89–2.03	.165	…			1.16	.79–1.69	.446	…		
Fungemia	2.99	.67–13.36	.152	…			0.90	.27–3.06	.872	…			1.12	.37–3.39	.837	…		
CSF																		
Culture sterile time >2 wk	0.96	.19–4.95	.960	…			0.92	.24–3.47	.899	…			0.92	.24–3.47	.899	…		
Glucose	0.996	.98–1.02	.722	…			0.99	.98–1.01	.486	…			1.00	.99–1.01	.699	…		
Protein	1.001	.9997–1.0025	.114	…			1.001	1.0002–1.0024	.**019**	1.0009	.9995–1.002	.189	1.001	1.0002–1.0024	.**018**	1.0008	.9995–1.002	.213
Leukocyte count	1.001	.999–1.004	.243	…			1.001	.998–1.004	.442	…			1.0009	.998–1.003	.505	…		
CrAg >1:512	1.03e+15	…	…	…			4.38	.57–33.6	.156	…			4.37	.57–33.7	.156	…		

Bold indicates *P* < .05.

Abbreviations: aHR, adjusted hazard ratio; CCI, Charlson Comorbidity Index; CrAg, cryptococcal antigen; CSF, cerebrospinal fluid; HR, hazard ratio; IICP, increased intracranial pressure.

## DISCUSSION

To the best of our knowledge, this is the first study conducted on the potential correlation between elevated CSF lactate levels and adverse outcomes in patients with CM who were HIV negative. Our findings indicate that, among individuals with CM, those without HIV exhibited higher CSF lactate levels than those with HIV. Notably, Abassi et al corroborated that CSF lactate levels exceeding 5 mmol/L were associated with early mortality in an HIV cohort [5]. In our cohort of patients with CM who were HIV negative, we established a link between CSF lactate levels exceeding 7.935 mmol/L and an increased risk of mortality between 30 and 90 days. This cutoff point exhibited exceptional specificity, suggesting that baseline lactate levels exceeding 7.935 mmol/L can effectively predict adverse outcomes in this patient population. Health care professionals can use this threshold to identify high-risk individuals early in their treatment course.

Lactic acid is the end product of glycolysis when pyruvate undergoes anaerobic metabolism in human cells. Elevated blood lactate levels in hypoxic conditions indicate inadequate tissue perfusion [11, 12]. The primary source of lactate in the CSF is glycolysis, which occurs in the central nervous system. In populations affected by infectious meningitis, elevated CSF lactate levels can be attributed to cerebral anaerobic metabolism associated with cerebral edema, reduced cerebral blood flow, inflammatory cell cytokines [13], and bacterial anaerobic glycolysis [11, 14, 15].

Previous observations in patients with tuberculous meningitis have shown that elevated intracranial pressure often occurs, leading to a significant decrease in cerebral blood flow when the intracranial pressure is uncompensated [16]. Lactate levels in ischemic brain cell tissues increase [17, 18], and lactate can spread from the ischemic brain tissue to the surrounding normal tissue, harming peripheral nerve cells, disrupting brain self-regulation, and worsening cerebral edema and ischemia [19]. Therefore, CSF lactate may serve as a potential prognostic factor [20, 21]. A rapid reduction in CSF lactate levels also indicates a favorable prognosis in patients with acute bacterial meningitis [22].

Furthermore, our study revealed that when compared with patients with CM who were HIV positive, patients with CM who were HIV negative tended to be older and have a higher prevalence of chronic comorbidities. This aligns with prior research findings suggesting that patients who are HIV negative may have specific risk factors that increase their susceptibility to cryptococcal diseases [23, 24]. For patients who were HIV negative, laboratory data from blood and CSF analyses revealed higher CSF WBC counts (including an elevated proportion of CSF WBC neutrophils), increased CSF protein levels, lower blood-CSF glucose ratios, and reduced fungal burdens in the blood. These differences may be attributed to the more intact immune response of patients without HIV than patients with HIV [24].

Patients who are HIV negative whose immune systems are unimpaired tend to have a more acute and robust inflammatory response to *Cryptococcus* [25, 26]. Panackal et al conducted a CSF analysis among patients with CM who were HIV negative and discovered elevated levels of neurofilament light chains, a biomarker indicative of axonal damage [25]. This increase suggests that the patient’s central nervous system cells experience severe axonal injury owing to an exaggerated host immune response following a serious infection. This underscores that an excessive inflammatory response can harm the human body [25]. Conversely, patients with HIV who have compromised immune functions typically exhibit milder and more chronic inflammatory responses to *Cryptococcus* infections, accompanied by a diminished capacity for fungal clearance [2]. Consequently, patients with HIV often exhibit higher rates of fungemia and greater fungal burdens in their blood and CSF than patients without HIV [2, 26, 27].

In addition to CSF lactate levels, CSF glucose [28, 29] and protein [28, 30] concentrations and a high fungal burden may serve as prognostic indicators for patients with CM [30]. CSF glucose levels ≤30.8 mg/dL [29] or failure to achieve a rapid decrease in CSF protein concentrations (a daily reduction of 0.18 mg/dL) following antifungal treatment may indicate a significant central nervous system inflammatory process with a poor prognosis in patients with CM [31, 32].

A baseline high CSF *Cryptococcus neoformans* count is associated with elevated intracranial pressure and higher mortality rates [33]. A rapid decrease in CSF *Cryptococcus* counts after 2 weeks of treatment in patients with CM who are HIV negative also signifies a good response to antifungal therapy [34]. CSF cryptococcal antigen is another indicator representing fungal burden in the CSF. A local study in Taiwan found that regardless of the HIV status of patients with CM, a high CSF antigen titer (>1:512) was associated with mortality at 10 weeks [35].

In the subsequent survival analysis, we found that all mortality cases occurred within 3 months of admission, accounting for 27.1% (19/70) of the total cohort. Considering mortality and neurologic sequelae as adverse outcomes, the overall adverse outcome rate 1 year after admission was 44.3% (31/70), with mortality accounting for 61.3% (19/31) of the adverse outcomes. It can be inferred that in patients with CM who develop neurologic sequelae, the early mortality rate within the first 90 days is relatively high. This high 90-day mortality rate may also result in death before the patient can receive an adequate duration of antifungal treatment, specifically among patients without HIV (Table 3). However, further investigation could not be conducted because of the limited sample size of our cohort.

Given that elevated CSF lactate levels can be used to predict mortality, they can also be used to predict the development of neurologic sequelae. Early mortality and permanent neurologic sequelae are likely to be interconnected aspects of this disease. Although CSF lactate level >7.935 mmol/L was a significant predictor of 90-day mortality in the univariate analysis, its impact was diminished in the multivariate regression analysis when additional variables were considered, likely due to the limited sample size. Consequently, larger multicenter studies are needed to validate these findings. This retrospective nonrandomized study from a single medical center in southern Taiwan over 30 years faced limitations, including its small sample size and potential variations in treatment strategies. Errors may have been introduced by using disease classification codes and manual record reviews. Future collaborations with domestic and international centers are planned for an extended prospective study to address these limitations.

## CONCLUSIONS

In our study, patients with CM who were HIV negative exhibited elevated CSF lactate levels, which correlated with mortality. This knowledge may enable health care professionals to identify high-risk individuals early in the treatment course. A possible further research area is the validation of the CSF lactate cutoff value of 7.935 mmol/L in a prospective population.

## Supplementary Material

ofae540_Supplementary_Data

## Supplementary Data

Supplementary materials are available at *Open Forum Infectious Diseases* online. Consisting of data provided by the authors to benefit the reader, the posted materials are not copyedited and are the sole responsibility of the authors, so questions or comments should be addressed to the corresponding author.

## Notes

***Acknowledgments*.** The authors thank the personnel at the Health Examination Center and Department of Medical Education and Research of Kaohsiung Veterans General Hospital for providing information in response to inquiries and for assistance in data processing. Y.-C. T. thanks his wife, Meng-Huang Chang, for her gracious thoughtfulness and invaluable assistance.

***Author contributions*.** Study design: Y.-C. T., Y.-S. C., C.-S. Y., R.-I. C., and J.-K. C. Data acquisition: Y.-C. T. and J.-K. C. Statistical analysis and data interpretation: Y.-C. T., Y.-S. C., C.-S. Y., R.-I. C., and J.-K. C. Drafting: Y.-C. T. Final revisions: Y.-C. T., Y.-S. C., C.-S. Y., R.-I. C., N.-C. W., and J.-K. C. All the authors have read and approved the final version of the manuscript.

***Availability of data and materials*.** All data generated or analyzed during this study are included in this published article.

***Financial support*.** This work was supported by the Veterans Affairs Council of Taiwan (grant VAC 113-001).
