# Supplementary material for: Cerebrospinal Fluid Lactate Levels as a Prognostic Indicator in Patients With Cryptococcal Meningitis Who Are HIV Negative: A Retrospective Cohort Study
Source: Open Forum Infect Dis. 2024 Sep 20;11(10):ofae540. doi: 10.1093/ofid/ofae540 (PMC11474979; doi:10.1093/ofid/ofae540)
Supplement: ofae540_Supplementary_Data [file ofae540_supplementary_data.docx]

Appendix 1. Patients treatment by group.

| Variables |  | Total  (n=70) | HIV-negative with CM (n=44) | HIV-positive with CM (n=26) | *p*-value |
| --- | --- | --- | --- | --- | --- |
| Amphotericin B + flucytosine | | 48(68.6) | 30(68.2) | 18(69.2) | 0.921 |
| Amphotericin B + fluconazole | | 22(31.4) | 14(31.8) | 8(30.8) | 0.927 |
| Liposomal Amphotericin B | | 5(7.1) | 3(6.8) | 2(7.7) | 0.891 |
| Fluconazole – maintenance | | 42(60) | 27(61.4) | 15(57.7) | 0.762 |

CM, cryptococcal meningitis; HIV, human immunodeficiency virus

Appendix 2. Radiographic findings

| Variables |  | Total  (n=70) | HIV-negative with CM (n=44) | HIV-positive with CM (n=26) | *p*-value |
| --- | --- | --- | --- | --- | --- |
| **Brain Image (CT or MRI)** | |  |  |  |  |
| Normal | | 33(47.1) | 17(38.6) | 16(61.5) | 0.064 |
| Meningeal enhance | | 12(17.1) | 10(22.7) | 2(7.7) | 0.188 |
| Hypodense lesions | | 18(25.7) | 14(31.8) | 4(15.4) | 0.163 |
| Hydrocephalus | | 15(21.4) | 10(22.7) | 5(19.2) | 0.730 |
| Acute infarction | | 6(8.6) | 5(11.4) | 1(3.9) | 0.401 |
| Old infarction | | 7(10) | 5(11.4) | 2(7.7) | 1.000 |
| Abscess | | 1(1.4) | 1(2.3) | 0 | 1.000 |
| Nodule | | 10(14.3) | 7(15.9) | 3(11.5) | 0.734 |
| **Chest films** | |  |  |  |  |
| Normal | | 36(51.4) | 21(47.7) | 15(57.7) | 0.420 |
| Nodule | | 12(17.1) | 8(18.2) | 4(15.4) | 1.000 |
| Mass | | 4(5.7) | 4(9.1) | 0 | 0.289 |
| Cavity | | 1(1.4) | 1(2.3) | 0 | 1.000 |
| Abscess | | 1(1.4) | 1(2.3) | 0 | 1.000 |
| Pleural involvement | | 5(7.1) | 5(11.4) | 0 | 0.074 |
| Infiltrate | | 22(31.4) | 13(29.6) | 9(34.6) | 0.791 |

CM, cryptococcal meningitis; CT, computer tomography; HIV, human immunodeficiency virus;

MRI, magnetic resonance imaging
